# Screening Food Insecure during Pregnancy: Pilot Testing an Effective Brief Tool for Use in an Australian Antenatal Care Setting

**DOI:** 10.3390/nu14214633

**Published:** 2022-11-03

**Authors:** Fiona H. McKay, Julia Zinga, Paige van der Pligt

**Affiliations:** 1 School of Health and Social Development, Institute for Health Transformation, Faculty of Health, Deakin University, Geelong, VIC 3220, Australia; 2 Royal Women’s Hospital, Parkville, VIC 3052, Australia; 3 The Institute for Physical Activity and Nutrition (IPAN), School of Exercise and Nutrition Sciences, Faculty of Health, Deakin University, Geelong, VIC 3220, Australia; 4 Department of Nutrition, Western Health, Footscray, VIC 3011, Australia

**Keywords:** food insecurity, pregnancy, screening, brief tool, clinical, antenatal

## Abstract

The purpose of this research is: (1) to determine the prevalence of food insecurity among pregnant people using the 10-, 6-, and 2-item iterations of the USA Household Food Security Survey Module (HFSSM) and the single item measure, and (2) identify an appropriate combination of questions that could be used to identify food insecurity in a clinical setting for a population of people who are pregnant in Australia. Cross-sectional survey collecting self-reported data from pregnant people in Australia (open May 2021 to March 2022). Survey included demographic characteristics, including income/welfare use, education, age, pregnancy information, household size and composition, and two measures of food insecurity. In total, 303 participants were included in the analysis. Sensitivity and specificity of the various combinations of questions were conducted. Food insecurity was estimated using the single item, and the 2-item, 6-item, and 10-item versions of the HFSSM, food insecurity was 6.2%, 11.4%, 11.7%, and 14.3% respectively. Respondents who were living in households that were food insecure answered affirmatively to question one, two, or three of the HFSSM, with the combination of questions one and three showing the best sensitivity and specificity for the whole sample, as well as for those who have characteristics likely to lead to food insecurity. Further testing of the 2-items, comprised of items one and three from the HFSSM, need to be conducted with a larger and more diverse sample to determine if this is an appropriate screening tool in an antenatal clinical setting to determine food insecurity during pregnancy.

## 1. Introduction

While food insecurity and hunger are known to have implications for health and wellbeing, households that experience food insecurity can be difficult to identify [1,2,3]. Food insecurity is characterised by limited access to safe, high-quality, and nutritious food, and the experience of food insecurity can prevent individuals from leading an active and healthy life [4]. While not all households will experience the same sequence of events or responses to food insecurity, food insecurity is considered to be a ‘managed process’ and a number of common responses at the household level have been identified [2]. According to Radimer and colleagues [5], when livelihoods are constrained, households first experience worry about securing sufficient food, they then employ a range of strategies to augment their food supply. If continued, they may compromise the quality of the food they consume, and finally, parents sacrifice the quantity of the food they consume, with children’s eating patterns disrupted only under very severe circumstances. This sequela of events has been reported in the literature [6,7], allowing some food insecure households to be identified through these characteristics.

There is currently no routine screening for food insecurity in Australian antenatal settings. However, there is a clear need to ensure food insecurity can be accurately and efficiently measured and responded to in a clinical setting. According to official government statistics, prevalence of food insecurity in Australian households is approximately 5% [8], however, this is generally considered an underestimate [9]. While starvation does not occur commonly in high income countries, children and adults do experience hunger, and chronic mild undernutrition can occur when financial resources are low [10]. Even short-term food insecurity and hunger can lead to mental and physical health implications and can impact learning, development, productivity, and family life [11]. While food insecurity can negatively impact health across the lifespan, it is especially problematic during pregnancy. Food insecurity and hunger during pregnancy is associated with increased risks of chronic conditions such as diabetes, hypertension, and cardiovascular disease, and is strongly linked with maternal overweight and obesity [12].

Many approaches to measuring food insecurity have been developed [13] and most build on the characteristics of food insecure households described above. One such measure, the US Household Food Security Survey Module (HFSSM), was developed to assess whether households have enough food or money to meet basic food needs and determine their behavioural and subjective responses [14]. The original HFSSM is comprised of 18 items, 8 of which are specific to households with children. The survey asks households if they worry about having the money to pay for food, whether there were times that the household was without food, and about whether specific household members went without food. Although widely used in research where it is considered the gold standard [15,16], the length of the survey and complex scoring algorithm limit routine clinical use. The 6-item HFSSM uses a subset of items from the 18-item HFSSM and is highly sensitive and specific, while this survey is commonly used in research, is also considered too long to be used in a clinical setting where time constraints limit such comprehensive scoring scales [17,18]. One or two item versions of the HFSSM have been used in clinical settings outside of Australia, and have been found to be sensitive and specific when assessing food insecurity among low-income families [19,20]. The Food and Agriculture Organization (FAO) of the United Nations has developed the Food Insecurity Experience Scale (FAO-FIES), an 8-item survey based on the HFSSM, that is used to assess experiences of food insecurity and compare population household food security status across countries [21], while shorter and somewhat easier to calculate, it is most commonly use at a population level use and in development settings [22,23]. In Australia, a recently developed tool for research use, the Household Food and Nutrition Security Survey (HFNSS) has been found to be a reliable and valid way to measure food insecurity at the household level [24]. At a population level in Australia, the Australian Health Survey employs the single-item measure of food insecurity that asks: “In the last 12 months was there any time you have run out of food and not been able to purchase more?” and has been used as an indicator for food insecurity [25]. Researchers generally point to this single item being an insufficient and inaccurate measure of food insecurity [9]. None of these tools have been explored as appropriate for use in antenatal care settings.

In recognition of the significant implications of food insecurity during pregnancy and the lack of a suitable measure for the identification of food insecurity in a clinical setting, the purpose of this research is twofold: (1) to determine the prevalence of food insecurity among pregnant people using the 10-, 6-, and 2-item iterations of the HFSSM and the single item measure, and (2) identify an appropriate combination of questions that could be used to identify food insecurity in a clinical setting for a population of people who are pregnant in Australia.

## 2. Materials and Methods

### 2.1. Recruitment

This cross-sectional study used a survey method to collect self-reported data from pregnant people in Australia. The survey was open between May 2021 and March 2022. The survey was widely published on social media (Facebook and Twitter), and flyers with a QR code linking to the online survey were available for people to take from clinic rooms at a large maternity hospital in Melbourne, a method based on previous research that was successful in recruiting this sample [26]. Only people who were pregnant (self-reported) and in Australia were able to participate. Ethics approval was obtained from Human Research Ethics Committees at the Royal Women’s Hospital and Deakin University (Protocols: Project 21/30 and 2021-344).

### 2.2. Data Collection

The consent procedure and all data were collected via an online survey, hosted by Qualtrics. The survey included 40 items, including demographic characteristics, including income/welfare use, education, age, gestational age (in weeks), number of previous pregnancies, and household size and composition (including number of children). Two measures of food insecurity were employed. (1) The 10-item HFSSM (the 18-item survey without the questions specifically related to children) to explore the overall experience of household food insecurity by investigating uncertain, insufficient, or inadequate food access, availability, and utilization [16], and (2) the single-item measure frequently incorporated into Australian population level health surveys which have assessed food insecurity [25] (see Table 1 for the breakdown of the questions included in each measure).

### 2.3. Data Analyses

Survey data were analysed using SPSS version 28 (SPSS, Inc., Armonk, NY, USA). Descriptive statistics were used to generate frequencies and proportions for categorical data (reported as *n* (%)) and means and standard deviations were generated for continuous data (reported as mean ± SD). Responses from the HFSSM were combined to create one score for level of food security for a household [16]. The food security status of each household was determined by the number of food insecure conditions and behaviours that the household reported. For the 10-item HFSSM, households were classified food secure if they report no food insecure conditions. Food insecure households were further classified as having marginal food security if they reported one or two food insecure conditions, low food security if they report between three and five food insecure conditions, or very low food security if reported six or more food insecurity conditions. Analyses were also conducted for the 6-item HFSSM, where households were classified food secure if they report no food insecure conditions, marginally food secure if they reported one condition, low food security if they reported two to four conditions, and very low food security if they reported more than five conditions. Analyses for the 2-item HFSSM were conducted with households considered to be food insecure if they report one or more condition.

We sought to identify if a specific and sensitive brief tool, using questions from the HFSSM, could be used to determine food insecurity among pregnant people with known risk factors for food insecurity. To determine if a brief measurement tool could be used to assess food insecurity of pregnant people, ideally in a clinical setting, cross tabulation was used to calculate specificity (the ability to correctly identify food-secure households), and sensitivity (the ability to correctly identify food-insecure households), using the 10-item HFSSM, where an individual provided 3 affirmative responses, as the reference measure consistent with previous research [20]. The prevalence of affirmative responses for each item on the HFSSM was calculated for the total sample (see Table 1). Prevalence data were used to generate sensitivity and specificity tables for combinations of two questions with the highest prevalence of affirmative responses among food insecure pregnant people. Like previous clinical screening tools for food insecurity [20,27], for each combination, an affirmative response to either question was considered an indicator of food insecurity, as there is little risk associated with misidentifying a patient as food insecure, and there is evidence to suggest that adverse health implications begin well before an individual or household reach the severe hunger stage [28]. Standard definitions of sensitivity, specificity, and accuracy were used [29]. Chi-square, Fisher’s Exact tests, and independent samples t-tests were used to determine associations between sociodemographic variables and food security status based on the 10-, 6- and 2-item HFSSM, and the single item from the survey (see Supplementary Material, Table S1). Data from the single item are provided in ‘yes/no’ format, households were considered food secure (negative response) or food insecure (affirmative response). Data from the HFSSM are provided as often true, sometimes true, never true, with often true and never true coded as 1, and never true coded as 0.

## 3. Results

Of the 449 surveys that were attempted, 66 were excluded as the respondent did not consent to the study or no questions were answered, 29 were excluded as they were currently not pregnant, 51 respondents were excluded as they did not answer one or more questions about food insecurity, leaving 303 participants with complete data who have been included in the analysis. For details on the sample and the factors that may contribute to food insecurity in this population please see [30,31]. Food insecurity was estimated using the single item and the 2-item, 6-item, and 10-item versions of the HFSSM, with the levels of food insecurity 6.2%, 11.4%, 11.7% and 14.3%, respectively, (see Table 2). Stratifying this further into levels of severity, the 10-item and 6-item estimated that 8.3% and 5.9% of the sample reported marginal food security, respectively, and that 6.3% and 5.9% reported low or very low food security. The single item and the 2-item tools are unable to distinguish level of food insecurity.

Most respondents who were living in households that were food insecure answered affirmatively to question one, two, or three of the HFSSM (see Table 1). These questions were combined to determine the sensitivity and specificity of the various 2-item combinations (see Table 3). Sensitivity and specificity were high for the whole sample, as well as for those who have characteristics which mean they are more likely to be food insecure [30,31]. Sensitivity ranged from 77.8% for items 1 and 2 for participants who already had children, to 100% for items 2 and 3, and 1 and 3 for people not living with their spouse. Specificity was higher and ranged from 88.2% for items 1 and 2 for participants who were not living with their spouse, to 100% for items 2 and 3, and 1 and 3 for the same population group within the sample. Accuracy was high (above 80%) for all 2 item combinations for all population groups. Overall, the combination of item 1 and 3 showed the highest sensitivity and specificity and is the recommended combination of questions to be used to determine food security in pregnant people.

## 4. Discussion

Food insecurity was identified using four measures of food insecurity, the single item used by the National Health Survey, and the 10, 6, and 2-item versions of the HFSSM. The single item provided the lowest estimates of food insecurity, with the 10-item version of the HFSSM providing the highest estimate. The 10-item version of the HFSSM is, like the 6-item, able to distinguish between different severities of food insecurity. The prevalence of food insecurity in this sample of pregnant people was 14.3% according to the 10-item version of the HFSSM. This is higher than the general community [8,9], and previous work exploring the prevalence of food insecurity during pregnancy in Australia [32]. This level of food insecurity is similar to recent research from Canada where 12.8% of 626 people who were pregnant were identified as food insecure [33], and similar to findings from the USA where 18.6% of 426 people who were pregnant were identified as food insecure [34]. Food insecurity is prevalent among pregnant women in Australia, demonstrating the urgent need for antenatal clinicians to have an efficient and effective food security screening tool which can be implemented in the current healthcare system in Australia. This will allow pregnant people to be supported throughout their pregnancy and to be able to attain optimum nutrition for best maternal and child health outcomes.

A 2-item food security screener, created by combining questions 1 and 3 of the 10-item USDA tool was found to be sensitive and specific, providing a quick and accurate method to identify food-insecure pregnant people. Specificity was high at 98.7% indicating that only 1.3% of households who were not food insecure were identified as such, while a sensitivity of 85.7% indicates that 14.3% of households that have experienced food insecurity were misclassified. Like other studies that have sought to determine a brief screening tool for a population [20,27], this study used the measurement of low or very low food insecurity as measured by the HFSSM, rather than any level of food insecurity as the reference measure for the sensitivity and specificity calculation. As such, it is possible that some participants were misclassified as food secure. However, supporting the validity of the 2-item screen suggested here, demographic groups that have been identified as more likely to experience food insecurity in other studies, including limited education, low income, and receipt of welfare [30,31] were found to have a higher frequency of food insecurity than other groups.

The results reported here are a first step in the creation and implementation of a screening tool that could be employed to identify food insecurity in an antenatal setting. Further testing in a variety of settings and with a diversity of population groups is needed. However, this study highlights that a 2-item tool, comprised of items 1 and 3 from the HFSSM, with polychotomous response options could be used in an antenatal clinical setting to determine food insecurity during pregnancy. This research recommends items 1 and 3, rather than 1 and 2 as employed in the 2-item HSFFM, as the combination of questions 1 and 3 demonstrated better accuracy, and reflect both worry about food shortages and cost pressures that have been identified as of concern for food insecure and hungry pregnant women [26,30]. This study indicates that the sensitivity and specificity of the 2-item screening tool means it could be used by antenatal clinicians to use to identify pregnant people who are food insecure. By limiting the screening tool to two items, this screen is short enough that it could be readily incorporated into electronic medical health records and used routinely for all pregnant patients in Australia.

## 5. Limitations

While there are clear outcomes of this study that are encouraging, there are several limitations that need to be taken into consideration. This study used the HFSSM 10-item as the reference for the sensitive and specificity testing, future work should consider validation against clinical conditions. Our study was limited in size, with just over 300 participants, this will affect the generalizability of our results. This is also not a representative sample, and future work should include more diversity in population, especially non-English speaking people who may have higher levels of food insecurity. Finally, there are limitations when proposing a two-item screening tool rather than the complete HFSSM as the shorter screening tool does not allow for the assessment of the severity of food insecurity. However, longer tools that are more commonly used for research purposes are not appropriate for use in busy clinical settings, nor do they take into consideration barriers including time constraints and increased workloads experienced by clinicians [17].

## 6. Conclusions

Food insecurity can have short- and long-term implications for both parent and child. Identifying a brief and valid screening tool that can be used in antenatal health care settings would be a positive step in the management of food insecure and hungry pregnant people for healthcare providers and will allowing for the implementation of a range of strategies to address hunger, food insecurity, and resulting poor nutrition. The rate of food insecurity identified in this study indicates an urgent need for antenatal clinicians to have an efficient and effective food insecurity screening tool which can be implemented in the current healthcare system in Australia. We recommend that brief screening tools for food insecurity be widely adopted in antenatal clinical settings.

## Figures and Tables

**Table 1:** Affirmative responses and items included in various food insecurity measures.

	HFSSM 10-Items	Affirmative Responses for Food Insecure Respondents	HFSSM 6-Item	Item 1 and 2/HFSSM 2-Item	Item 1 and 3	Item 2 and 3
1	I worried whether my/our food would run out before I/we got money to buy more.	73%		Yes	Yes	
2	The food that I/we bought just didn’t last, and I/we didn’t have money to get more.	45%	Yes	Yes		Yes
3	I/we couldn’t afford to eat balanced meals.	68%	Yes		Yes	Yes
4	In the last month, did you or other adults in your household ever cut the size of your meals or skip meals because there wasn’t enough money for food?	27%	Yes			
5	How often did this happen?	27%	Yes			
6	In the last month, did you ever eat less than you felt you should because there wasn’t enough money for food?	23%	Yes			
7	In the last month, were you ever hungry but didn’t eat because there wasn’t enough money for food?	27%	Yes			
8	In the last month, did you lose weight because there wasn’t enough money for food?	7%				
9	In the last month, did you or other adults in your household ever not eat for a whole day because there wasn’t enough money for food?	5%				
10	How often did this happen?	5%				

**Table 2:** Prevalence and level of food security measured by different tools (*n* = 303).

Prevalence	Single Item	2-Item HFSSM	6-Item HFSSM	10-Item HFSSM
Food secure (%)	93.8	88.6	88.3	85.7
Food insecure (%)	6.2	11.4	11.7	14.3
Level of food security				
Food secure	93.5	86.6	88.3	84.4
Marginal food security	NA	NA	5.9	8.3
Low or very low food security	NA	NA	5.9	6.3

**Table 3:** Sensitivity and Specificity of 2-item groupings (*n* = 303).

		Items 1 + 2			Items 2 + 3			Items 1 + 3		
	Prevalence *	Sensitivity	Specificity	Accuracy	Sensitivity	Specificity	Accuracy	Sensitivity	Specificity	Accuracy
All households	6.2	82.4	98.3	97.3	93.3	98.3	98.0	85.7	99.6	98.7
Households with children	7.8	77.8	98.1	82.3	88.9	99.1	91.1	100	98.1	99.6
Highschool or diploma	10.0	81.8	98.0	96.4	81.1	99.0	97.2	90.9	98.0	97.3
Not living with spouse	15.0	100	88.2	90.0	100	100	100.0	100	100	100.0
Income under 70,000	32.4	96.0	83.3	87.4	83.3	100	94.6	96.0	91.7	93.1
Welfare recipient	22.5	88.9	96.8	95.0	88.9	100	97.5	88.9	100	97.5

* Prevalence according to the HFSSM 10-item tool, 3 or more affirmative responses, used as the reference measure.

## Data Availability

The datasets generated during and/or analysed during the current study are not publicly available due to ongoing research and planned further analysis but are available from the corresponding author on reasonable request. A supplementary table with full detail of analyses are included.

## Supplementary Materials

The following supporting information can be downloaded at: https://www.mdpi.com/article/10.3390/nu14214633/s1, Table S1: Proportion of respondents who were food insecure for a range of demographic characteristics.
