# Supplementary material for: Screening Food Insecure during Pregnancy: Pilot Testing an Effective Brief Tool for Use in an Australian Antenatal Care Setting
Source: Nutrients. 2022 Nov 3;14(21):4633. doi: 10.3390/nu14214633 (PMC9654102; doi:10.3390/nu14214633)
Supplement: Supplementary file 1 [file nutrients-14-04633-s001.zip › nutrients-1998969-supplementary.pdf]

nutrients

MDPI

Article

Screening Food Insecure during Pregnancy: Pilot Testing an Effective Brief Tool for Use in an Australian Antenatal Care Setting

Fiona H. McKay [1,*], Julia Zinga [1,2] and Paige van der Pligt [3,4]

[1] School of Health and Social Development, Institute for Health Transformation, Faculty of Health, Deakin University, Geelong, VIC 3220, Australia
[2] Royal Women's Hospital, Parkville, VIC 3052, Australia
[3] The Institute for Physical Activity and Nutrition (IPAN), School of Exercise and Nutrition Sciences, Faculty of Health, Deakin University, Geelong, VIC 3220, Australia
[4] Department of Nutrition, Western Health, Footscray, VIC 3011, Australia
* Correspondence: fiona.mckay@deakin.edu.au; Tel.: +61-3-92517183

Table S1. Proportion of respondents who were food insecure for a range of demographic characteristics.

	Single item	2-item USDA	6-item USDA	10-item USDA
**Food insecure % (n)[1]**	6.2 (19)	11.4 (35)	11.7 (36)	14.3 (44)
**Gestation**				
First trimester	16.7	23.5	20.6	21.4
Second trimester	50.0	47.1	41.2	42.9
Third trimester	33.3	29.4	38.2	35.7
**Previous pregnancies**				
0	52.6	55.9	55.5	54.8
1+	47.7	44.1	38.8	45.2
**Maternal age**				
18-24	22.2*	18.2*	19.4	15.9
25-29	26.3	28.6	25.0	27.3
30-34	15.8	22.9	30.6	29.5
35+	31.6	25.7	19.4*	22.7*
No response	5.3	5.7	5.6	4.5
**Annual income (AUD)**				
0-20,000	10.5*	5.7	5.6	4.5
20,001-50,000	21.1*	28.6*	27.8*	25.0*
50,001-70,000	15.8*	8.6	11.1	9.1
70,001-90,000	15.8	17.1*	13.9	13.6*
90,001-120,000	26.3	20.0	30.6*	27.3*
120,000+	10.5*	14.3*	11.1*	15.9*
No response/prefer not to say		2.9		4.6
Receiving welfare	36.8*	37.1*	33.3*	34.1*
**SEIFA**				
Low	33.3	29.4	34.3	32.6
Middle	27.8	35.3	34.3	34.9
High	38.9	35.3	31.4	32.6
**Country of birth**				
Australia and New Zealand	84.2	68.6	75.0	70.5

**Living situation**				
Spouse/partner	78.9	77.1*	83.3*	81.8*
Alone	5.3	8.6	5.6	6.8
Parents/family/friends	10.5	11.4*	8.3*	9.1*
**Education**				
Year 12 or less	10.5	14.3*	11.1	11.4
Diploma or graduate diploma	57.9*	40.0	50.0*	43.2*
Bachelor degree	15.8	22.9	30.6	27.3
Postgraduate degree	15.8	20.0	8.3*	15.9*
**Children in the home**				
0	42.1	48.6	52.8	50
1	31.6	31.4	33.3	31.8
2+	21.1	17.1	11.1	15.9*
No response	5.2	2.9	2.8	18.2

**p <.05 significantly different to other responses in this indicator

[1] n= number of respondents who were identified as food insecure using that measure
